# Intestinal microbiota modulates neuroinflammatory response and brain injury after neonatal hypoxia-ischemia

**DOI:** 10.1080/19490976.2024.2333808

**Published:** 2024-03-27

**Authors:** Alexander Drobyshevsky, Sylvia Synowiec, Ivan Goussakov, Rafael Fabres, Jing Lu, Michael Caplan

**Affiliations:** a Department of Pediatrics, NorthShore University HealthSystem, Evanston, IL, USA; b Department of Pediatrics, Pritzker School of Medicine, University of Chicago, Chicago, IL, USA

**Keywords:** Gut-brain interaction, neuroinflammation, hypoxia-ischemia, perinatal brain injury

## Abstract

Premature infants lack a normal intestinal microbial community and also at risk of perinatal hypoxic-ischemic (HI) brain injury, which is considered to be one of the major factors for motor, sensory, and cognitive deficits. We hypothesized that neonatal gut microbiota composition modulated the immune reaction and severity of neonatal H-I brain injury. Neonatal C57BL/6J mouse pups were exposed to H-I protocol consisting of permanent left carotid artery ligation, followed by 8% hypoxia for 60 min. Microbial manipulation groups included 1) antibiotic treatment, E18 (maternal) to P5; 2) antibiotic treatment E18 to P5 + E. coli gavage; 3) antibiotic treatment E18 to P5 + B. infantis gavage; and 4) saline to pups with dams getting fresh water. The extent of brain injury and recovery was measured on MRI. Edematous injury volume was significantly higher in E. coli group than that in B. infantis group and in fresh water group. Gene expression in brains of pro-inflammatory cytokines (IL1β, IL6, IL2, TNF-α and toll-like receptors 2–6) were elevated to a greater extent in the E. coli group at P10, no injury, and at P13, 72 hours after H-I relative to sham control and B. infantis groups. Significant effects of microbiome and brain injury and interaction of these factors were found in abundance of major phyla. The neuroinflammatory response and brain injury after neonatal hypoxia-ischemia are affected by intestinal microbiota, providing opportunities for therapeutic intervention through targeting the early colonization and development of the gut microbiota.

## 1. Introduction

During the perinatal period, microbes from the mother and the surrounding environment colonize the gastrointestinal tract of infants, until a dense, complex bacterial community is established. Premature infants are exposed to a hostile extra-uterine environment and to unnatural microbiota in the hospital that results in abnormal intestinal colonization.^1^ In addition, premature infants often lack normal commensal bacterial communities, due to frequent abdominal deliveries and lack of maternal breast feeding and skin-to-skin interaction. Colonization of beneficial bacteria such as *Lactobacilli* and *Bifidobacteria* is often delayed in preterm infants and is only found in relatively low numbers during the first few weeks of life, whereas potentially pathogenic bacteria such as *E. coli*, *Clostridia*, and *Staphylococci* are found in higher numbers.^2,3^ Considering that one of the most pronounced effects of gut microbiota is on maturation of the infant immune system,^4^ variations in immune responses in premature infants may reflect the influence of abnormal gut microbiota.^5^

Preterm infants, particularly those born at less than 32 weeks post-conception age and/or with a birth-weight less than 1500 g are at risk for adverse neurological outcomes, including cognitive and behavioral deficits.^6,7^ The most important risk factors leading to neurologic deficits in the newborn period include hypoxia and maternal inflammation.^8,9^ Inflammation in neonatal brain and unbalanced immune responses play a pivotal role in the extent of damage and recovery after perinatal hypoxic-ischemic (H-I) brain injury.^9–11^ It has been suggested that an unbalanced, accentuated pro-inflammatory response in neonates, associated with an impaired anti-inflammatory capability,^12^ plays a major role in brain injury.^11,13^

Perinatal hypoxic–ischemic brain damage is a major cause of acute mortality and chronic neurologic morbidity in infants and children and often is associated with inflammation. Inflammation occurs before, during, and after the brain injury, and modulates the vulnerability to and development of brain injury.^9^ The effects of inflammation and ischemia are likely modulated by early gut microbial composition through immune, endocrine, and neural pathways.^14^ A prime example of the interaction between gut dysbiosis and brain injury in premature infants is necrotizing enterocolitis (NEC).^15^ The key features of the disease include formula gavage, hypoxia episodes, and TLR4 signaling.^16^ Survivors of NEC suffer brain volume loss, demyelination and develop profound neurological impairments.^17^ Specific probiotics, including Bifidobacterium, were effective in preventing NEC in very-low-birth-weight infants.^18^

Considering the link between gut microbiome, systemic inflammation, and hypoxia, we hypothesized that neonatal gut microbiota modulates the extent of brain injury and recovery after hypoxic-ischemic insult via inflammatory pathways. To test this hypothesis, we manipulated gut microbial composition in an established murine model of perinatal H-I. We created several scenarios of neonatal gut dysbiosis with bacterial depletion by maternal antibiotic administration, followed by gavage transfaunation with either pathogenic or commensal bacteria and examined the relationship between the inflammatory response and the severity of brain injury and recovery.

## 2. Results

We examined the effect of microbial manipulation on neonatal mouse gut by maternal antibiotic treatment, followed by a gavage of either Escherichia coli (“E. coli” group), Bifidobacterium longum infantis (“B. infantis”), or sterile saline (“Antibiotics”) and fresh water controls, on H-I brain injury and neuroinflammatory response.

### 2.1. Spatial distribution and temporal evolution of brain edema after H-I injury

**Figure 1.**
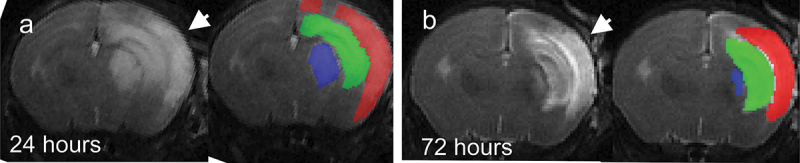
Temporal evolution of brain injury after neonatal mouse H-I injury on serial T2-weighted MR images. Hyperintensity regions (arrowheads), indicative of edema, were apparent on serial imaging at 24 (A) and 72 h (B) after H-I insult. Hyperintensity regions were manually outlined in cortex (red), hippocampus (green), and striatum (blue). B.

Unilateral H-I resulted in brain edema that could be unambiguously delineated on T2-weighted MR images as hyperintense areas at 24 and 72 h after H-I insult (Figure 1).

The severity of H-I brain injury on MRI was stratified by hyperintense volume as 1) no injury (0 mm^2^), 2) moderate (<30 mm^2^), and 3) severe (>30 mm^2^). Between 4 and 22% of animals in our study did not exhibit apparent changes on T2-weighted images after H-I, representing minimal to no injury. The occurrence of cases with no injury on MRI was not different between the microbiota treatment group (χ2 = 2.593, df = 3, *p* = 0.459). A pairwise comparison of outcome groups revealed a smaller proportion of animals in the severe group in the B. infantis group relative to the E. coli group at 24 and 72 h (*p* = .0036 and *p* = .0016, Fisher’s exact test). No other significant differences in severity outcome on MRI were found.

In summary, we observed that the distribution of the severity of brain injury was typical for this neonatal mouse H-I model^19^ and found a decrease in the proportion of animals with severe brain injury in the B. infantis group.

### 2.2. H-I edema volume was the largest in E. coli and the smallest in B. infantis groups

**Figure 2.**
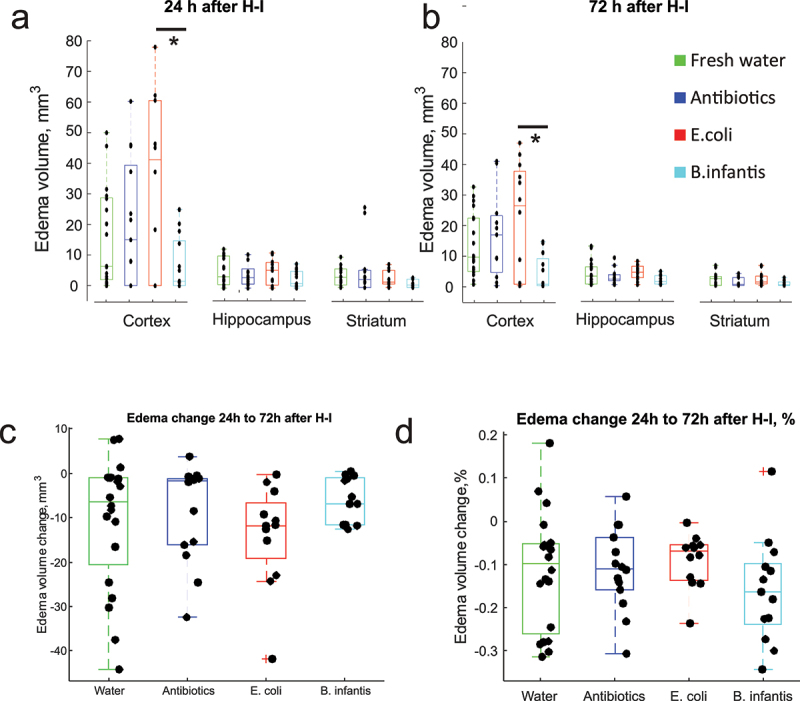
Edema volumes at 24 and 72 h after H-I. A,B – regional edema volume in cortex, hippocampus and striatum. C,D. Change of edema volume from 24 to 72 h after H-I. No difference was found between microbiota treatment groups in absolute or relative amount of edema volume change. *- significant difference between group means (ANOVA with Tukey-Kramer post-hoc tests).

The analysis of variance showed that the effect of microbiota treatment on total edema volume was significant at 24 h, F(3,55) = 5.03, *p* = .0037, and at 72 h after H-I, F(3,55) = 6.07, *p* = .0012. The total volume of edema was significantly larger in E. coli group relative to *B. infantis* group on post-hoc analysis at 24 (*p* = .0015) and at 72 h (*p* = .0005) and relative to the Fresh water group at 72 h (*p* = .048). There was no significance in total edema volume between the other groups. When compared between brain regions, the decrease of edema in *B. infantis* group relative to *E. coli* group was significant only in cerebral cortex: F(3,55) = 4.11, *p* = .011 at 24 h and F(3,55) = 3.88, *p* = .014 at 72 h (Figure 2a, b).

In summary, we observed a larger edema volume in E. coli group, predominantly due to an injury to the cerebral cortex.

### 2.3. Brain recovery after H-I injury between 24 and 72 h was not different between microbiota groups

Serial MRI measurements revealed that the edematous brain volume decreased between 24 and 72 h in most cases (Figure 2e, f). The initial H-I cellular injury, manifested as edema at 24 h, may resolve without tissue loss. However, hyperintense areas on T2-weighted images at 72 h represent the tissue with irreversible injury that will predominantly undergo necrosis, as confirmed previously by histology.^20^ There was no difference between the microbiota treatment groups in edema volume change in absolute units (one-way ANOVA F(3,55) = 1.03, *p* = . 38), or when the change is expressed as a fraction of initial edema volume (one-way ANOVA F(3,55) = 0.58, *p* = .65).

### 2.4. Gene expression of pro-inflammatory cytokines increase in E. coli group brains at P10, no injury, and at P13, after H-I

We examined the gene expression of pro- and anti-inflammatory cytokines in the whole brain tissue with real-time PCR on separate cohorts on animals at two time points: P10, no injury and at P13, 72 h after H-I injury.

**Figure 3.**
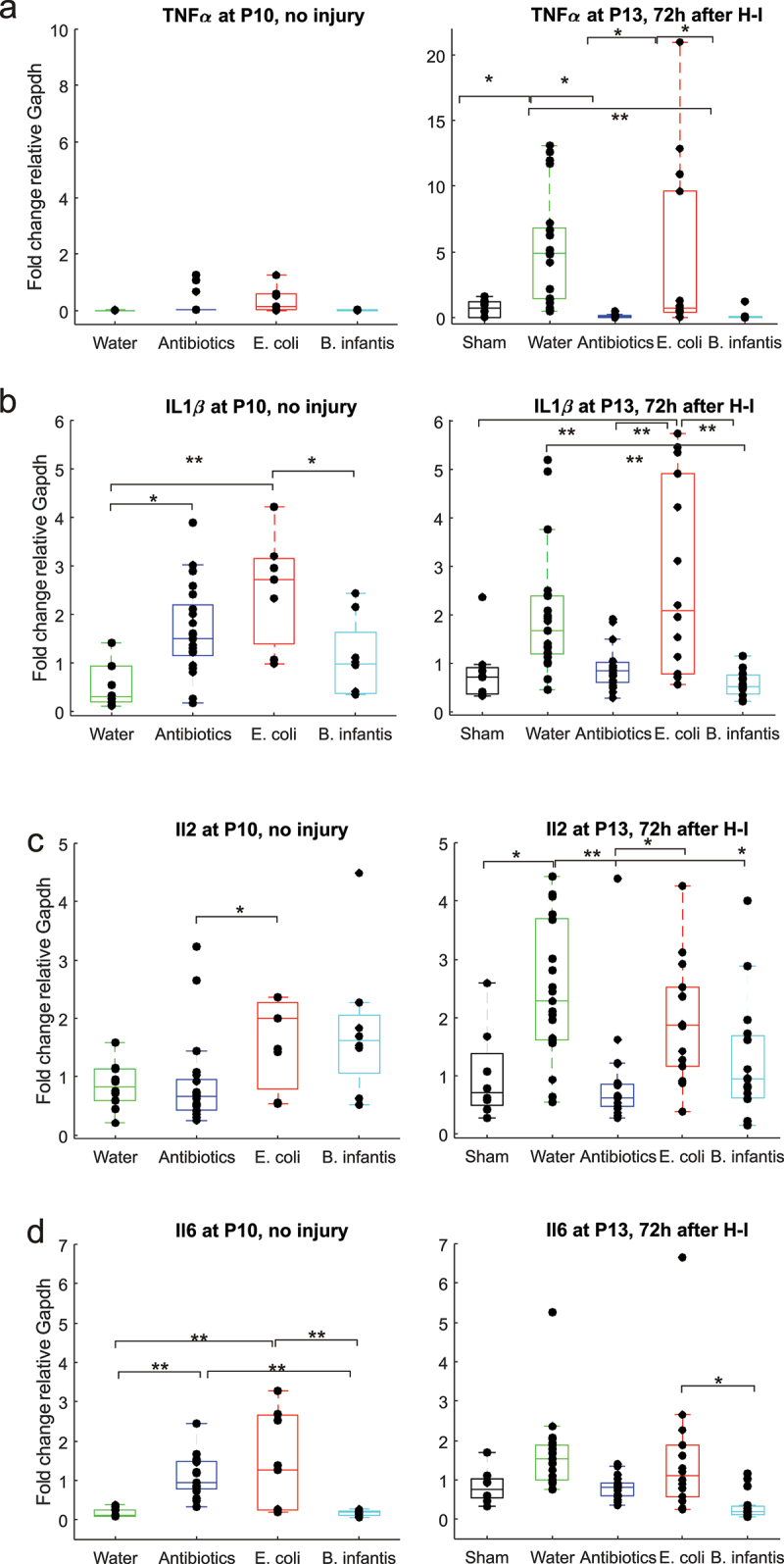
Pro-inflammatory cytokines gene expression before and after H-I across microbiota treatment groups. *-p < 0.05, ** - *p* < 0.01 on post-hoc tests.

At P10, expression of pro-inflammatory cytokines TNFα was not different between microbiota groups (F(3,42) = 2.37, *p* = .083, Figure 3a). At P13, 72 h after H-I, expression of TNFα was higher in Fresh water and *E. coli* groups (F(4,63) = 4.63, *p* < .001) relative to the sham control group. Noticeably, TNFα expression did not change with H-I in *B. infantis* and Antibiotics groups.

The effect of microbiota treatment on IL1β gene expression was significant at P10, F(3,42) = 7.75, *p* = 3.13e-04. IL1β expression was elevated in Antibiotics and in *E. coli* groups (Figure 3b). At P13, 72 h after H-I, the effect of microbiota treatment on IL1β gene expression was also significant (F(4,63) = 9.04, *p* < .001). IL1β expression was elevated in *E. coli* group relative to sham and Fresh water group, while it was lower in *B. Infantis* group relative to Fresh water group.

At P10, the expression of pro-inflammatory cytokine IL2 was higher in *E. coli* groups relative to Antibiotics group (F(3,42) = 4.09, *p* < .01, Figure 3c). At P13, 72 h after H-I, expression of IL2 was higher in fresh water and *E. coli* groups (F(4,63) = 9.11, *p* < .001). At P10, expression of pro-inflammatory cytokine IL6 was higher in Antibiotics and *E. coli* groups relative to Fresh water and *B. infantis* groups (F(3,42) = 11.33, *p* < .001, Figure 3d). At P13, 72 h after H-I, expression of IL6 was higher in *E. coli* group relative to *B. infantis* group.

A two-way ANOVA revealed that there was a statistically significant interaction between the effects of gut microbial treatment and H-I factors for all examined pro-inflammatory cytokines expressions: TNFα (F(3, 107) = 6.05, *p* < .001), IL1β (F(3, 107) = 6.25, *p* < .001), IL 2 (F(3, 107) = 6.25, *p* < .000), IL6 (F(3, 107) = 4.48, *p* < .001). Simple main effects of the microbial treatment modulated the expression of proinflammatory cytokines TNFα (F(1, 107) = 15.39, *p* < .001) and IL6 (F (1, 107) = 3.13, *p* = .031) after H-I with a large, 2- to 3-fold, increase in Fresh water and *E. coli* groups (Figure 3).

In summary, microbiota manipulation altered the expression of pro-inflammatory cytokines at P10, with a significant increase in the E.coli group. After H-I, at P13, the expression of the pro-inflammatory cytokine was increased relative to the age-matched sham controls, in Fresh water and E.coli group, but not in Antibiotics and B.infantis group.

### 2.5. Anti-inflammatory cytokines gene expression increase in E.Coli group before and after H-I

**Figure 4.**
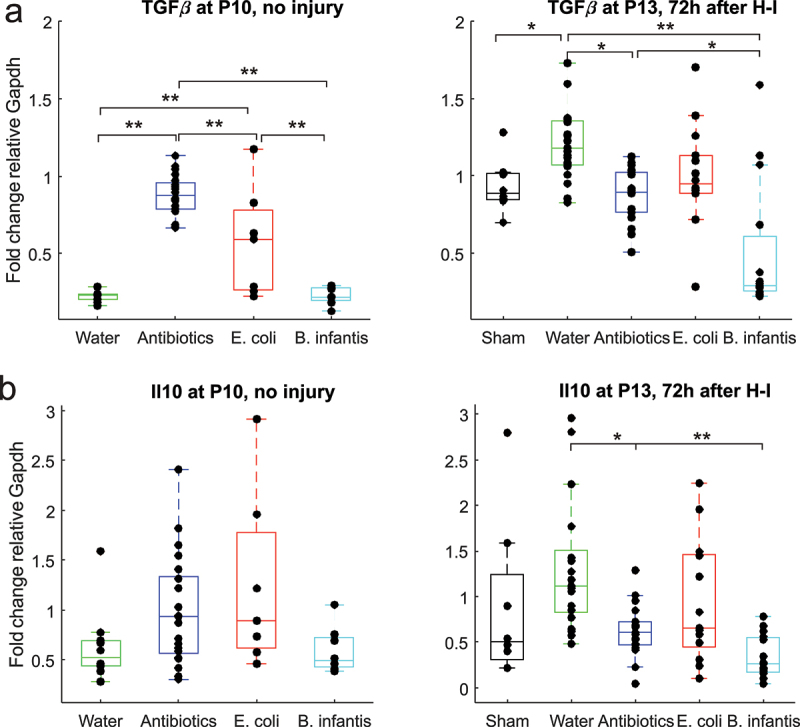
Anti-inflammatory cytokines gene expression before and after H-I in different microbiota treatment groups. *-p < 0.05, ** - *p* < 0.01 on post-hoc tests.

At P10, ANOVA revealed that the effect of microbial treatment on expression of anti-inflammatory cytokine TGFβ was significant (F (3,42) = 50.8, *p* < .001, Figure 4a), with increased expression of TGFβ and Antibiotics groups in post-hoc analysis. At P13, 72 h after H-I, expression of TGFβ was decreased in the Antibiotics and *B. infantis* groups relative to Fresh water (F(4,63) = 10.26, *p* < .001).

At P10 ANOVA revealed that the effect of microbial treatment on expression of anti-inflammatory cytokine IL10 was significant (F(3,42) = 2.85, *p* = .048, Figure 4b), but there was no difference between the group on post-hoc analysis. At P13, 72 h after H-I, expression of IL10 was decreased in Antibiotics and *B. infantis* groups relative to Fresh water (F(4,63) = 5.75, *p* < .001).

A two-way ANOVA revealed that there was a statistically significant interaction between the effects of gut microbial treatment and H-I for all examined anti-inflammatory cytokines expressions: TGFβ (F(3, 107) = 19.7, *p* < .001), IL10 (F(3, 107) = 6.11, *p* < .001), indicating that the increased anti-inflammatory cytokines with H-I was only in Fresh water and *E. coli* groups. Simple main effects of H-I factor were significant for expression of TGFβ (F(1, 107) = 62.36, *p* < .001), indicating increase of TGFβ expression after H-I, but was not significant for IL10 (F(1, 107) = 0.24, *p* = 0.62).

In summary, we observed an increased expression of anti-inflammatory cytokine at P10 and P13 relative to the Fresh water group except B.infantis group.

### 2.6. Microbial treatment contributed to pro-inflammatory cytokine expression independently of initial injury after H-I

**Figure 5.**
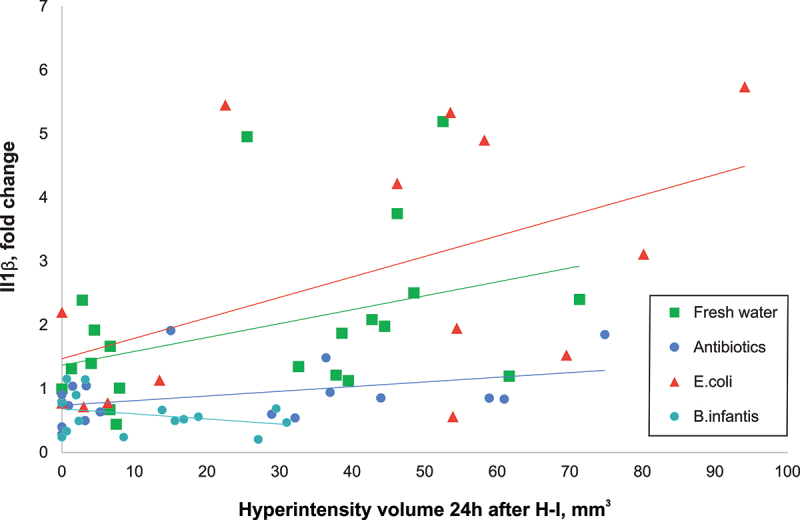
Pro-inflammatory cytokine IL1β increased proportionally with the extent of H-I injury. The linear regression slopes of the increase were significantly lower in antibiotic group relative to fresh water and E. coli groups, and even lower in B. infantis group.

Cytokine expressions increased with the volume of injury after H-I (Figure 5). To examine whether microbiota composition contributes to the neuroinflammatory reaction independently of injury severity, gene expression of cytokines was modeled using ANCOVA with injury volume at 24 h as a covariate. Coefficients of the ANOVA for each studied cytokine are presented in Supplementary Table S1. The *E. coli* group had the largest positive, and *B. infantis* had the largest negative significant intercepts in the linear models, which explains the levels of pro-inflammatory IL1β independently of the extent of H-I brain injury.

**Figure 6.**
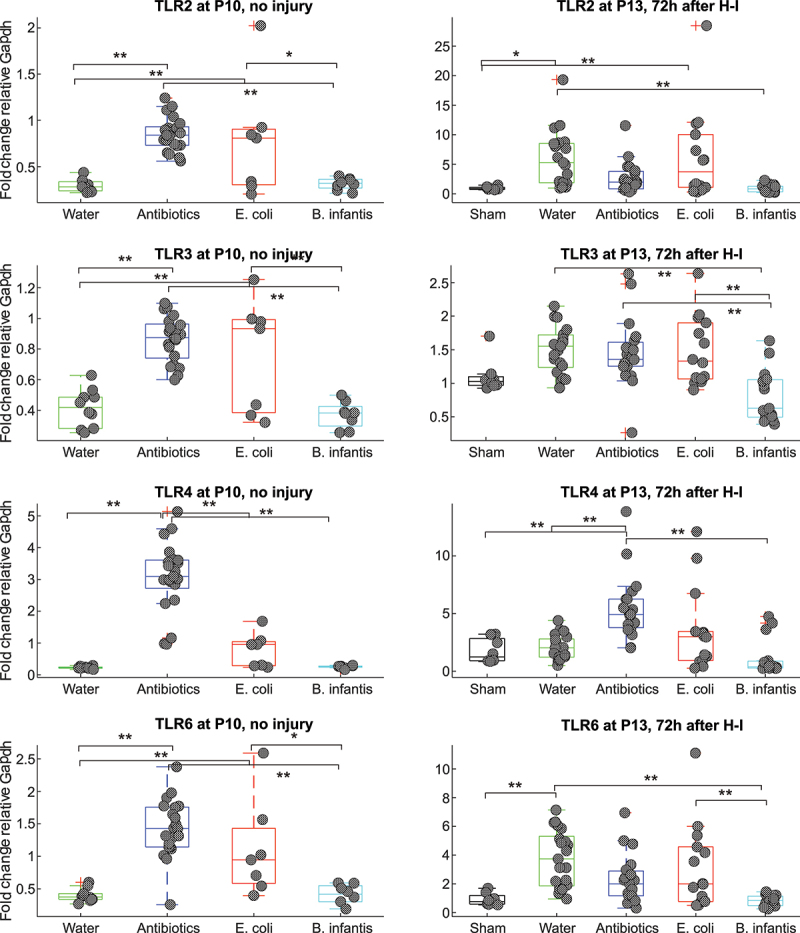
Gene expression of TLRs before and after H-I in different microbiota treatment groups. *-p < 0.05, ** - *p* < 0.01 based on post-hoc tests.

Two-way ANOVA analysis revealed that both H-I and microbiota treatment and their interaction were significant factors for all TLR expressions, as summarized in Supplementary Table S2. Expression of all studied TLRs in brains before H-I was increased in Antibiotics and *E. coli* groups relative to the Fresh water and *B. infantis* groups (Figure 6). After H-I, the expression of all TLRs increased several folds relative to pre-H-I values and shams in Fresh water, Antibiotics and *E. coli* groups without difference between the groups. Remarkably, the expression of TLRs in *B. infantis* group remained on the same level as in shams, approximately at pre-H-I levels.

In summary, cytokine expressions increased with the volume of injury after H-I, with the largest slope in the E.coli group.

### 2.7. Activation of astroglia and microglia activation after H-I is decreased in B. infantis groups

**Figure 7.**
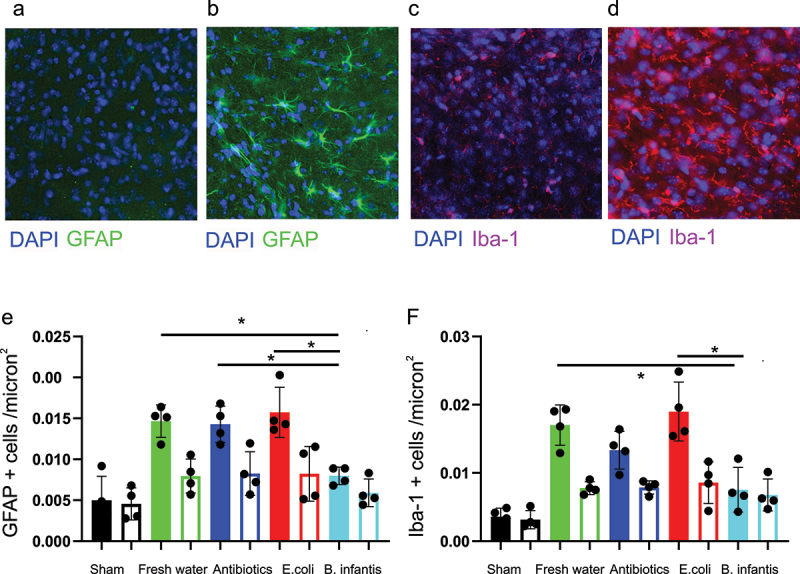
Activation of astro glia and microglia activation after H-I.

To test whether the differential expression of brain cytokines after H-I in microbiota groups corresponded to the level of reactive gliosis, which is known to mediate neuroinflammatory response,^21^ brain sections were stained for GFAP and Iba-1 markers to identify activated astro- and microglia cells (Figure 7). The number of GFAP and Iba-1 positive reactive glia cells was very low in sham controls (Figure 7a, c), there was a dramatic increase in the number of reactive glia cells in the hemisphere ipsilateral to the carotid ligation (Figure 7b, c). Microbial treatment was a significant factor in the number of reactive astrocytes (F(3, 12) = 10.15, *p* = .0013), and activated microglia cells (F(3, 12) = 8.89, *p* = .0022), Figure 7e, f. Post-hoc Tukey’s multiple comparisons test revealed significantly smaller number reactive astroglia in the B.infantis group relative to the Fresh water (*p* = .005), Antibiotic (*p* = .0077), and E.coli (*p* = .0016) groups. Similarly, the number of activated microglia cells were lower in B.infantis group relative to the Fresh water (*p* = .0087) and E.coli (*p* = .0016) groups. The number of reactive glia cells was significantly lower in the cortex contralateral to the carotid ligation side, but slightly higher than in the sham, with no difference between microbiota groups.

A. Representative images of GFAP and Iba-1 immunofluorescence for each experimental group in the cortex cerebral area ipsilateral to carotid occlusion at P13, 72 h after H-I. Density of reactive astroglia (E) and activates microglia (F) in cerebral cortex in ipsilateral (filled bars) and contralateral (empty bars) hemisphere. *- significant difference between group means, ANOVA with Tukey-Kramer post-hoc tests.

In summary, reactive astro- and microgliosis was observed in the ipsilateral hemisphere in all microbial treatment groups and was significantly smaller in the B.infantis group.

### 2.8. Small intestines injury after H-I

**Figure 8.**
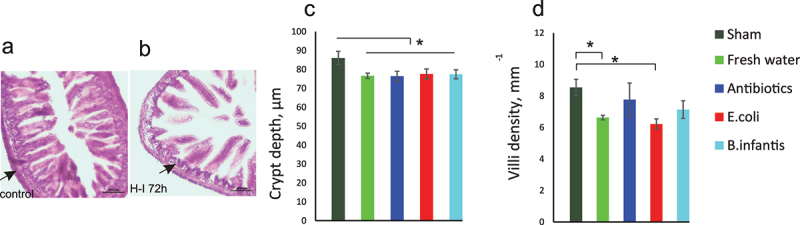
Small intestine injury after hypoxia – ischemia. Hematoxylin and eosin staining of terminal ileum of controls (A) and mouse pups in the fresh water group 72 h after H-I (B). Decreased thickness of lamina propria is indicated by arrows. Crypt depth (C) and villi density (C) were decreased after H-I in all microbiota groups. *- *p* < 0.05 post-hoc group comparisons with control group.

To examine whether microbial manipulation and neonatal gut dysbiosis may result in a selective gut susceptibility to hypoxia during H-I insult, terminal ileum morphology was examined on H&E staining. Small intestine injury after H-I was evident by structural changes (Figure 8a, b). Crypt depth decreased at 72 h after H-I in all microbiota groups relative to sham controls (one-way ANOVA F(4,24) = 3.30, *p* = .031, Figure 8c). This decrease was not different between microbiota groups. Villi density was lower in the Fresh water and *E. coli* groups after H-I relative to the sham controls (Figure 8d, f(4,24) = 9.04, *p* < .001). No difference after H-I was found in villi length between the microbiota groups (F(4,24) = 0.79, *p* = .51).

In summary, injury in small intestines was observed in all microbiota groups after H-I.

### 2.9. Serum pro-inflammatory cytokines increase after global hypoxia without brain injury in all microbiota groups

To test whether global H-I injury to organs other than brain contributed to the increase in serum cytokine levels independently of brain injury, we subjected all microbial manipulation groups to 8% hypoxia protocol at P10 but did not perform carotid artery ligation surgery. With this regimen, we expected no brain injury, confirmed by the absence of cell injury in non-ligated ipsilateral hemisphere in a similar neonatal hypoxia-ischemia mice model.^22^ Animals were euthanized and serum cytokines concentrations were measured in mice subjected to global hypoxia in age-matched normoxia controls.

**Figure 9.**
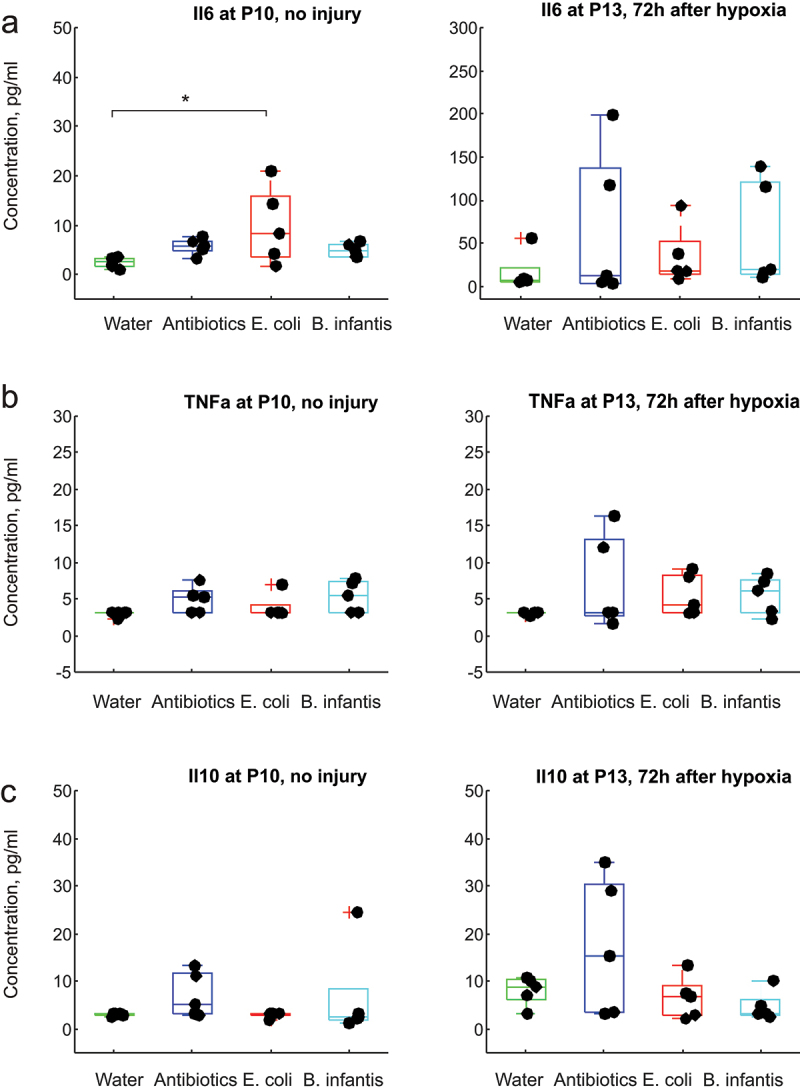
Cytokines serum concentrations before and after global hypoxia without carotid ligation. *-p < 0.05, ** - *p* < 0.01 on post-hoc tests.

Serum concentrations of IL1β, IL2, IL4, and interferon-γ, with and without hypoxia, were lower than the ELISA assay sensitivity (<3 pg/mL). A one-way ANOVA revealed a significant effect of microbial treatment on expression of pro-inflammatory cytokine IL6 in the group without hypoxia (F(3,17) = 3.22, *p* = .042, Figure 9), with the increase of IL6 in *E. coli* relative to Fresh water group (post-hoc *p* = .031). No difference between microbiota treatment groups was found in IL6 after hypoxia (ANOVA F(3,16) = 0.81). No difference between microbiota treatment groups was found in serum concentration of TNFα before hypoxia (F(3,17) = 2.31, *p* = 0.11) or after hypoxia (F(3,16) = 1.02, *p* = 0.40) as well as in serum concentration of IL10 before (F(3,17) = 0.95, *p* = 0.43) and after hypoxia (F(3,16) = 2.49, *p* = .09).

A two-way ANOVA revealed a significant effect of hypoxia on serum concentration of IL6 (F(1,33) = 9.56, *p* = .004), resulting in elevated IL6 in all microbiota groups except fresh water, Figure 9a. No other significant effects of microbiome treatment or hypoxia or interaction between them were found in pro-inflammatory TNFα and anti-inflammatory IL10 (Figure 9b, c).

In summary, the level of circulating cytokines was increased after global H-I without brain injury that may be an independent factor determining neonatal brain injury severity.

### 2.10. Changes in neonatal gut microbiome composition with transfaunation and after H-I

**Figure 10.**
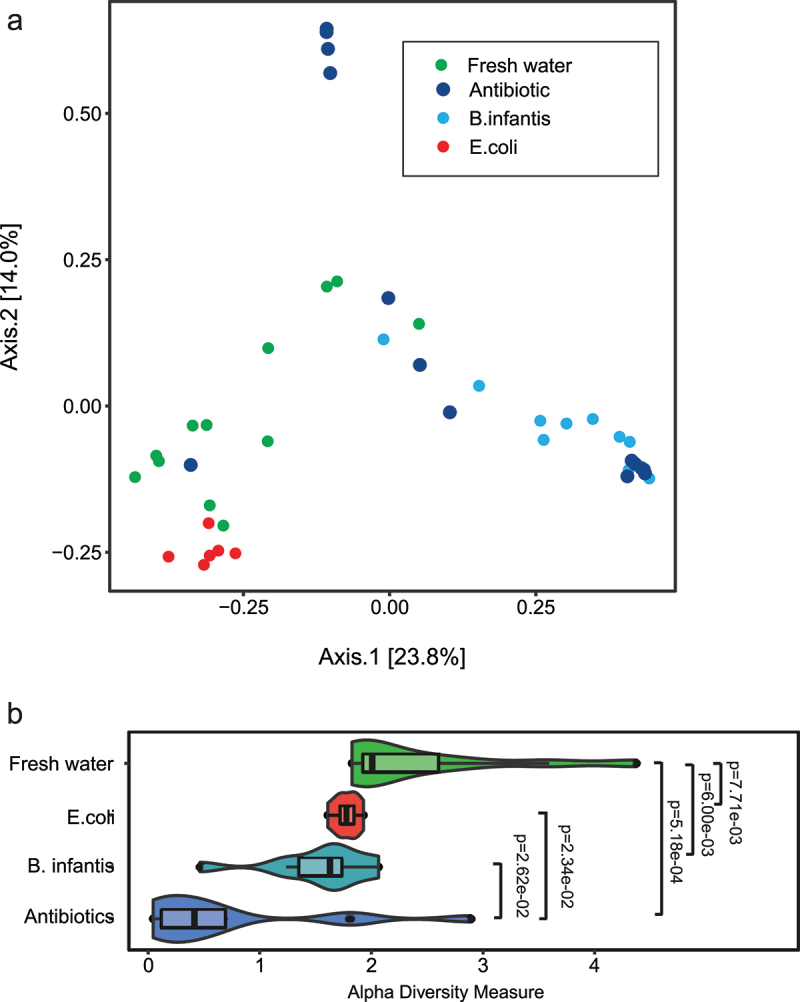
Indexes of mouse pup microbiome biodiversity after neonatal gavage transfaunation using 16S rRNA gene sequencing before H-I at P10. A. Beta diversity PCoA plots with bray-curtis dissimilarity measure, showing separation of gut bacterial communities with different microbial treatment. B. Shannon index of alpha-diversity was significantly lower in the Antibiotics group relative to fresh water group, but no difference between E. coli and B. infantis groups.

Indexes of mouse pup microbiome biodiversity were examined using 16S rRNA gene sequencing of colon microbiota at P10, no injury and at P13, 72 h after H-I. There was a distinct separation of *E. coli* and water groups using principal component analysis with Bray-Curtis dissimilarity measure (Figure 10a, ANOSIM, *R* = 0.762; *p* = .001), while the *B. infantis* and antibiotics groups had a large overlap. The Shannon index of alpha-diversity was significantly lower in Antibiotics group relative to Fresh water group, indicating the successful depletion of microbiota in neonatal digestive tract after maternal antibiotic treatment until P10. Alpha-diversity increased with microbial transfaunation with *E. coli* and *B. infantis* by P10 after the maternal antibiotics treatment but did not reach the level of the Fresh water group.

**Figure 11.**
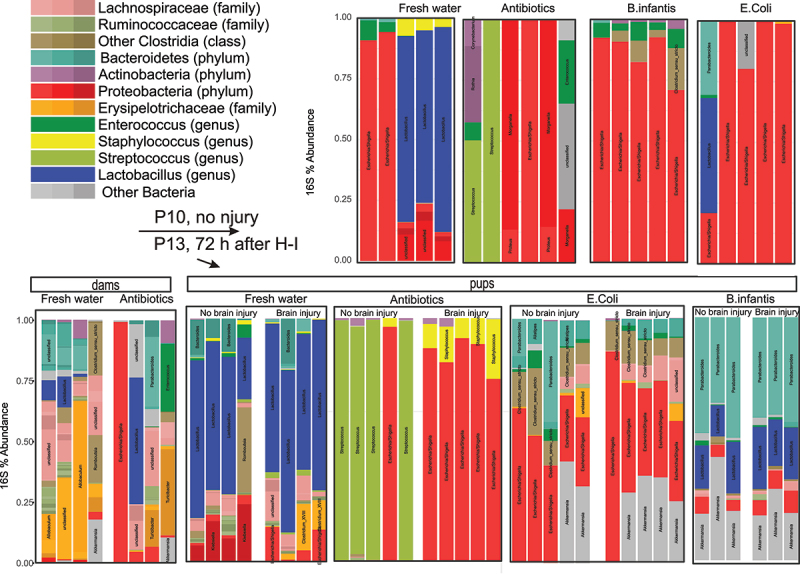
Taxonomic distribution in OTUs in colon samples obtained from mouse pups before (P10) and after IH (P13). The samples after H-I were sorted onto by the extent of brain injury.

Microbial composition was apparently different between microbiota treatment groups at P10, no injury, as shown on the distribution of operational taxonomic units (OTU) in large intestine samples (Figure 11). Fresh water controls had a large abundance of Lactobacillus genus relative to the other microbiota treatment groups. By P13, all mice, except Antibiotics group, showed an increase of diversity in 16s abundance, similar to the previously described trend in neonatal gut microbiome development.^14^ We also separated groups with and without brain injury to examine possible interactions. After the insult, pups with brain injury in Antibiotics group had a larger proportion of Escherichia/Shigella family. Pups in *B. infantis* group had a larger proportion of Bacteroidetes and Lactobacillus (Figure 11).

**Figure 12.**
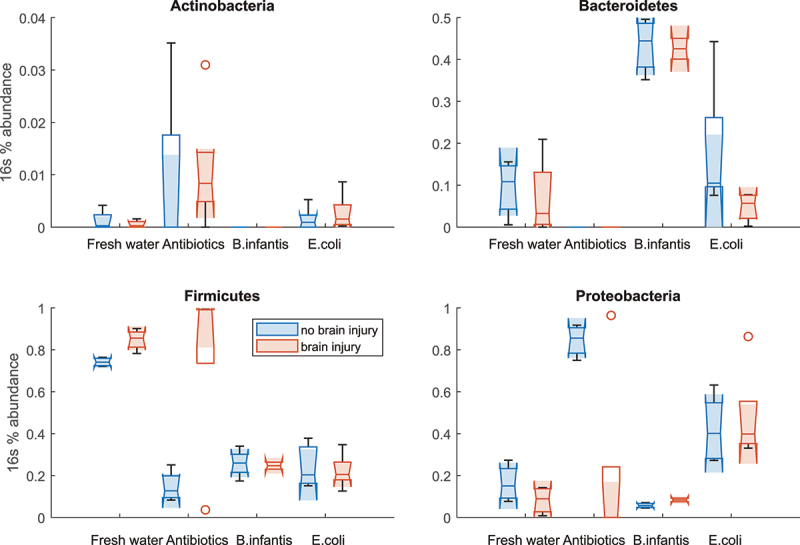
16s abundance for the four most abundant phyla in colon samples in pups with and without brain injury at P13 after H-I.

We further analyze OTU abundance changes between microbial treatment groups in pups with and without brain injury on MRI in four major phyla (Figure 12). Two-way ANOVA revealed no effects of microbiome and brain injury on Actinobacteria (Figure 12a) and a significant main effect of microbiome F(3,25) = 35.7, *p* < .001 in abundance of Bacteroidetes phylum (Figure 12b). The relative abundance of Bacteroidetes was significantly higher in *B. infantis* group regardless of brain injury factor. Two-way ANOVA revealed significant main effects of microbiome F(3,25) = 16.41, *p* < .001 and brain injury factors F(1,25) = 7.52, *p* = .01, and their interactions F(3,25) = 6.52, *p* = .002 in abundance of Firmicutes (Figure 12c). Significant main effects of microbiome F(3,25) = 8.97, *p* < .001 and brain injury factors F(1,25) = 4.59, *p* = .04, and their interactions F(3,25) = 5.48, *p* = .004 were also found in abundance of Proteobacteria (Figure 12d). The significant effect of brain injury factor was driven primarily by with higher Firmicutes and low Proteobacteria in antibiotic group.

In summary, we observed a complex interaction between H-I insult, brain injury, and microbial composition in neonatal guts.

## 3. Discussion

The major finding of the study was that neonatal gut microbiota modulated the extent of brain injury and neuroinflammatory response after H-I in a neonatal mouse model. Transfaunation of mouse pups early after birth with pathogenic bacteria E. coli significantly increased the H-I-induced brain injury relative to saline control pups from dams receiving fresh water. The extent of brain injury in pups, transfaunated with commensal bacteria B. infantis, remained similar to the saline control. Expression of proinflammatory cytokines in the brain was higher in E. coli group before and after H-I and was associated with the larger H-I brain injury, while B. infantis significantly decreased extent of H-I injury and lowered pro-inflammatory cytokines before and after H-I. This increase of pro-inflammatory cytokines occurred in conjunction with elevated expression of TLR in brains of E. coli groups. Elevated pro-inflammatory cytokines closely corresponded to the level of reactive astro- and microgliosis and was significantly smaller in the B.infantis group. We did not find evidence that gut microbial composition differentially affect pro- and anti-inflammatory cytokine increase after global hypoxia without brain injury. Microbial manipulation and brain injury were also significant factors affecting microbiota gut composition, indexes of biodiversity and relative abundance of major bacterial phyla in neonatal intestines at 72 h after H-I.

In the current study, we determined that the exposure to opportunistic pathogenic (E. coli) or commensal (B. infantis) bacteria modulated inflammatory responses before and after hypoxic-ischemic brain injury. Bacteria strains were chosen to provide pro- (E. coli) and anti-inflammatory (B. infantis) effects on brain based on published data. Both taxa are common both to human and conventional mouse gut flora and typically present in hospital environment. E. coli is associated with necrotizing enterocolitis (NEC) in premature infants.^15^ Inoculation of germ free parent mice with enteropathogenic E. coli increased pro-inflammatory cytokines IL1β and IL6 in plasma of offspring.^16^ Transfaunation with B. infantis increased the expression anti-inflammatory cytokines IL-10 and TGF-β1 in mouse colon tissue,^17^ and decreased IL1β in a rat colitis model.^17^ Bifidobacterium is the dominant bacterial genus in normal neonatal intestinal flora (commensal bacteria)^18,23^ and has received attention for preventing NEC.^24^ Bifidobacterium strains increased anti-inflammatory cytokines IL-4, IL-10 in inoculated germ-free mice^25^ and improved cognition in BALB/c mice.^26^

In neonates, systemic modulation of inflammation has been shown to affect the extent of brain hypoxic-ischemic injury.^27,28^ Bacterial exposure and systemic inflammation in neonates have been modeled with lipopolysaccharide (LPS) injections, which dramatically increased H-I brain injury.^28^ Sensitization or protective preconditioning to H-I depended on the timing of LPS injection: brain injury was decreased if LPS had been administered 24 h before H-I and increased in a shorter or longer period before H-I.^29^ In our study, we observed an increase of pro-inflammatory cytokines in blood and in brains in E. coli group, associated with an increased injury volume on MRI, suggesting primary sensitization of neonatal brains to H-I due to change in neonatal gut microbial composition. E. coli group had also increased TLRs expression in brains before H-I, suggestive of elevated bacterial component presence in the pups’ systemic circulation.

There was a large, several fold increase of TLRs 2, 3, 4, and 6 and proinflammatory cytokines TNF-α, IL1β, IL2 gene expression in brains after H-I, in accordance with previous reports.^30,31^ Expression of the proinflammatory cytokine was proportional to the extent of injury, but higher for the E. coli group compared to the Fresh water group given the same amount of the injury. Markedly, transfaunation with B. infantis resulted in the least amount of brain injury and expression of TLRs and pro-inflammatory cytokines before and after H-I across all groups, which suggests a neuroprotective effect of this microbiota manipulation due to decreased sensitization and inflammatory response to H-I. Taken together, we have demonstrated that proinflammatory E. coli is associated with increased brain injury and neuroinflammation before and after H-I injury, whereas commensal B. infantis had protective effects on brain injury and decreased neuroinflammation after H-I.

We examined whether the differential expression of pro- and anti-inflammatory cytokines was related to changes in the intestinal wall integrity due to dysbiosis caused by microbiota manipulations or due to global hypoxia injury to organs other than brain, including intestines. Both scenarios are relevant to clinical conditions of dysbiosis and exposure to intermittent hypoxic episodes in preemies or birth asphyxia in term infants and may result in damage to intestines.^32^ We did find histopathological evidence of structural injury in ileum after H-I, including crypt depth and villi density decrease in all microbiota groups relative to Fresh water sham controls, especially in E. coli group. At the same time, the level of circulating pro-inflammatory cytokine IL6 was elevated in the experiment of global hypoxia without brain injury, indicating that hypoxic injury to the other organs may contribute independently to H-I brain injury. Possible mechanisms could involve inflammation and activation of HIF and other molecules after global hypoxia that can have an impact on cytokine expression, possibly in gut tissue, but in other sites as well.^33^

Microbiota manipulations in this study altered the normal pattern of microbial colonization in the intestine of neonatal mice, that is typically characterized by a large presence of Lactobacillus.^34^ Maternal antibiotics administration dramatically reduced bacterial load and biodiversity of intestinal microbiome, as reported before.^14^ The presence of a complex and diverse intestinal flora was found to be functionally important for regulating intestinal mucosal and systemic immune responses^35^ and provide resistance to viral and bacterial infections, including E. coli.^5,14^ While transfaunation with E. coli and B. infantis in our study increased alpha diversity measures compared to Fresh water controls, the composition of the microbiota was different between these groups, which may explain the difference in the extent of H-I injury and magnitude of neuroinflammatory response. We also found significant effects of microbiota composition and H-I brain injury on 16s abundance of major phyla, suggesting that H-I injury in the brain affects microbial composition in the bowl, as observed in adult mice,^36^ but this observation requires additional data.

Our study included maternal antibiotic administration until P5 to model dysbiosis in offspring. While antibiotics were essentially eliminated from the pups’ systemic circulation before H-I insult and were unlikely to directly affect the neuroinflammatory response to H-I as demonstrated in post-H-I insult antibiotic administration,^27,37^ they had a long-lasting effect by depleting microbiota and dramatically reducing microbiome biodiversity. The situation of developmental dysbiosis is clinically relevant since infants are often treated with systemic antibiotics.^38^ While mouse pups after maternal antibiotics, followed by saline gavage in our study, demonstrated a decrease of pro-inflammatory cytokines IL1b, IL2, TNF-α, and also significant increase of TGFβ in brains after H-I, there was no difference in initial injury size or the dynamics of injury size in this group relative to fresh water controls. Therefore, early antibiotic treatment resulting in microbiota depletion and low gut microbial biodiversity did not reduce susceptibility of infant brain to H-I injury, but may affect the long-term recovery.

The study has several limitations and over simplifications. The experimental groups defined by microbial manipulation were intended to model conditions of “pathogenic versus commensal” bacteria load, which is likely an over simplification. E. coli indeed is a normal component of neonatal gut microbiota and may be beneficial, along with other bacteria, including Bifidobacterium, to the maturation of normal antibody response to infection.^39^ However, E. coli is known as an opportunistic pathogen, and its presence in large amounts has been associated with necrotizing enterocolitis in premature infants.^15^ Transfaunation of E. coli in large amounts in the immature mouse intestines in our study was intended to create dysbiosis with increased systemic inflammation.^16^

Second, the assessment of neuroinflammation before and after H-I insult at was conducted at different time points, P10 and P13, on separate cohorts of animals. However, there was no significant difference found between the Fresh water group at P10 and the Fresh water group with sham surgery at P13, indicating minimal confounding effect of age on expression of the studied cytokines and justifying the use of 2-way ANOVA to examine the effect of H-I and microbiota factors using two age groups.

Third, TGF-β was referred to as an anti-inflammatory cytokine, as commonly refereed in the perinatal neuroinflammation field,^11^ although it has both a pro- and an anti-inflammatory action, participating in the resolution of chronic neuroinflammation.^9^

Fourth, antibiotics from the mother’s milk may be absorbed in the pups’ intestine and affect the response to H-I insult. However, we expected that the systemic concentration of antibiotics in pups to decrease to minimal values during 6 days from cessation of maternal antibiotic administration at P5 before H-I insult at P10, and do not provide anti-inflammatory and neuroprotective effect as described in rodent models with antibiotics administration around the time of H-I.^27,37^

Fifth, we did not use a carotid ligation normoxia group. Carotid ligation alone (without hypoxia) in the Vannucci model in mice and rats does not cause permanent cell death.^8^ Instead, the confounding inflammatory effects of the surgery were controlled in the sham group where incision was made but the vessels were not manipulated.

In adults, the microbiome is now considered as an established modulator of secondary neuroinflammation and regeneration after experimental stroke^40^ and in human patients.^41^ In adult mice, microbiota modulation with antibiotics was protective against stroke via a mechanism that was dependent on IL-17.^42^ Multiple mechanisms linking microbiota as well as the associated metabolites have been proposed to play a role in pathogenesis of ischemic stroke, including oxidative stress, apoptosis, and neuroinflammation.^43^ The current study examined the role of the microbiome in the regulation of neuroinflammation, but the other factor affecting infant brain susceptibility to hypoxia-ischemia due to gut-brain interaction remains to be discovered. Infant microbiota is increasingly recognized as a critical contributor to brain cognitive development.^44–47^ Simple strategies, like oral administration of specific probiotics (such as B. infantis) may be neuroprotective to decrease the volume and risk of brain damage after perinatal hypoxic ischemic brain injury.

## 4. Materials and methods

**Figure 13.**
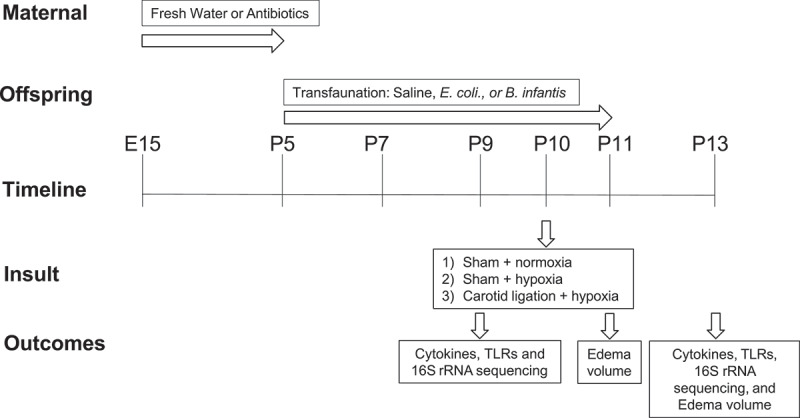
Time line of the experiments and experimental groups. The study groups are formed by a combination of two experimental factors: 1) microbial manipulation (fresh water, saline, E.Coli, B. infantis) and 2) hypoxia (normoxia, hypoxia, carotid ligation + hypoxia), at 2 time points. Each group had the same outcome measures, listed in the bottom row.

Timeline of the experiments is shown in Figure 13 and experimental groups and sample numbers are in Supplementary Table S3.

### 4.1. Manipulation of intestinal microbiota in neonatal mice

C57BL/6J mice were originally obtained from Jackson Laboratory (Bar Harbor, Maine) and bred in house. Time pregnant mice were maintained in autoclaved plastic cages with autoclaved food and water. Starting day E15 of pregnancy (5 days before term delivery) mice received sterile drinking water mixed with five antibiotics: (ampicillin, gentamicin, metronidazole, vancomycin, neomycin, all 1 mg/ml, from Sigma-Aldrich). This regiment has been shown to deplete bacterial load in neonates by at least a magnitude of 16sRNA copies/mg of intestinal content.^14^ Depletion of neonatal gut microbiota mimics abnormal gut flora development that may occur after cesarean section or antibiotic application in neonates and also equalizes the colonization opportunities with microbial manipulations. Antibiotic water was changed weekly. The dams continued to receive antibiotic containing drinking water after the pups were born until P5. After P5, sterile drinking water was given to the dams until the experiment was finished.

At P5, littermates were randomly assigned to microbial treatment groups and received a gavage suspension of either *Escherichia coli* (“*E. coli*” group, *n* = 28), or *Bifidobacterium longum infantis* (“*B. infantis*” group, *n* = 23), or sterile saline (“Antibiotics” group, *n* = 44). Naïve pups in the control group were fed by dams receiving fresh water (“Fresh water” group, *n* = 37) throughout the experiment. The bacteria were obtained from the American Type Culture Collection (*E. coli* ATCC 10,798 and *B. infantis* ATCC 15,697). Bacteria were plated on agar plates and selected colonies free of contamination were propagated in tryptic soy broth type E. The bacteria concentration was measured using spectrophotometer (Siemens Micro Scan Turbidity Meter). All vials with bacteria were adjusted to reach the 0.5 Mcfarland units that approximates 1.5 × 10^8^ cell count density per 1 mL, by adding more bacteria cells with a cotton swab. The vials were spun down; pellets with cells were collected and stored in −80°C until use. Before each feeding, cells were re-suspended and each pup received 100 µL of gavage suspension containing 1.5 × 10^8^ CFU of bacteria or saline at P5, P7, P9, and P11 using a silicone gavage tube.

Fecal samples of the pups were collected from the pups’ colons at P10 and P13 (before and after H-I insult, correspondingly) and stored at −80°C for sequencing analysis. The intestinal contents were processes at the University of Chicago Duchossois Family Institute facility for genomic DNA extraction and subsequent 16S rRNA gene sequencing on the Illumina MiSeq platform. Data processing was conducted using R statistical software version 3.6.2 and the R package DADA2 version 1.14.1 pipeline.^48^ Specifically, reads were first trimmed at 180 bp for both forward and reverse read to remove low-quality nucleotides. Chimeras were detected and removed using the default consensus method in the DADA2 pipeline. Then, ASVs with lengths between 300 and 360 base pairs were kept and deemed as high-quality ASVs. Taxonomy of the resultant ASVs wase assigned to the genus level using the RDP database with a minimum bootstrap confidence score of 50. Multiple sequence alignment of all ASVs was performed to generate a neighbor-join phylogenetic tree using the R package “msa” (v1.18.0) and “ape” (v5.3). Indexes of microbiome alpha (Shannon) diversity or overall β-diversity were calculated.

### 4.2. Neonatal brain hypoxia-ischemia

At age P10, neonatal mice of both sexes from each litter were subjected to H-I insults using the Vannucci model adapted for neonatal mice.^22,49^ Briefly, the left carotid artery was permanently ligated with a double knot silk suture 7–0 under isoflurane anesthesia. Lidocaine was added to the wound for local analgesia. The wound was sutured, and pups returned to dam for recovery and nursing for 3 h, followed by 60 min of hypoxia with 8% O_2_ at 37°C. Temperature during H-I was maintained by a servo-controlled heating blanket with temperature probe inside hypoxic chamber (Homeothermic Blanket System, Harvard apparatus, MA). Sham controls from the Fresh water group underwent surgery with carotid artery exposed but not ligated and did not receive hypoxia. Mortality rate due to the surgical manipulation and hypoxia was between 11.8–18.2% and not different between microbiota treatment groups. Therefore, experimental groups were “Fresh water +H-I”, *n* = 31, “”Antibiotics + H-I”, *n* = 44, E.coli+H-I”, *n* = 28, “B.infantis + H-I”, *n* = 23, “Fresh water +sham H-I”, *n* = 6.

### 4.3. Neonatal global hypoxia

To evaluate the systemic inflammatory response to hypoxia independently of brain injury, a separate set of sham operated animals with carotid artery exposed but not ligated (*n* = 4 in each microbiota group, i.e. “Fresh water”, “Antibiotics”, E.coli”, “B.infantis”) underwent 60 min of hypoxia with 8% O_2_ at 37°C. There was no mortality in this experiment. Blood samples were obtained 4 h after hypoxia exposure using carotid artery incision under isoflurane anesthesia and spanned down. Serum was frozen for cytokine measurements by multiplex ELISA.

### 4.4. Injury volume on MR imaging

At 24 and 72 h after H-I, serial MRI data were acquired for all pups. During the imaging, mice were anesthetized with 1% isoflurane in air and kept warm on a heated water blanket. MR images were acquired on a 9.4 Tesla scanner (Bruker Biospin, Billerica, MA) using a Bruker mouse receive-only surface coil and a 72 mm quadrature transmitter coil. T2-weighted images were acquired using a fast spin echo RARE sequence with TE/TR 40/3000 ms, 4 signal averages, echo train length 8, in-plane resolution 0.12 × 0.12 mm. Twenty coronal slices with thicknesses 0.7 mm were placed to cover the whole brain. To increase throughput, pups were scanned in pairs. Hyperintense areas indicating injured brain regions (edema) were manually outlined on each slice using ITK-SNAP 3.2 (itksnap.org). Volumes of edematous areas in the cortex, hippocampus, striatum, as well as total edematous volumes were calculated.

### 4.5. RNA isolation and real-time PCR

Only the animals with hyperintense regions, detected either on T2-weighted MRI at 24 h or 72 h (Supplementary Table S3), were included in the further analysis after H-I: Fresh water group, *n* = 16, Antibiotics group, *n* = 14, *E. coli* group, *n* = 13, *B. infantis* group, *n* = 8. Brain and ileum tissues were collected and individually frozen on dry ice and stored at −80°C before use. The ipsilateral (left) brain hemisphere tissue was homogenized in Qiazol Lysis Reagent (cat. # 79306 Qiagen, Germantown, MD) on ice for extraction of total RNA. NanoDrop (ThermoFisher Scientific, USA) instrument was used to measure RNA quantity and quality. 500 ng of isolated total RNA was used to synthesize cDNA using an RT2 First Strand Kit from QIAGEN. cDNA was amplified by polymerase chain reaction (PCR) with Quantitect Sybr Green PCR Kit (Qiagen, Germantown, MD, cat. # 204143) on an Applied Biosystems GeneAmp 5700 real-time quantitative PCR instrument. The total reaction volume of 25 μL consisting of 1.0 ul RT product (cDNA). Primers for TLR2–6, IL1b, IL2, IL6, IL8, IL10, TNF-α, TGFβb, GAPDH for Sybr Green Assay were predesigned and ordered from Integrated DNA Technologies, Inc., USA. For each primer, the final concentration was 0.4 μm and annealing temperature was adjusted. Gene expression was normalized to the housekeeping gene GAPDH and expressed as a fold change of experimental controls using delta-delta Ct method.

### 4.6. Inflammatory cytokine measurements by multiplex ELISA

Blood serum samples were used for cytokine analysis. Protein levels of cytokines TNF-α, IL-1β, IL-2, IL-6, IL-10, interferon-γ were assessed using a MILLIPLEX® Mouse Cytokine/Chemokine magnetic bead panel (MCYTOMAG-70, Millipore) according to the manufacturer’s instructions. Standard curves were generated using the specifics standards supplied by the manufacturer. Samples were analyzed on a MAGPIX® system (Millipore) using the MILLIPLEX® Analyst 5.1 software (Millipore).

### 4.7. Histological assessment of glia activation in brains after H-I

Pups were euthanized at P13, 72 h after H-I by transcardial perfusion with saline solution (0.9% NaCl) followed by 4% paraformaldehyde (PFA). Brains were removed and post-fixed in 4% PFA overnight, soaked in 30% sucrose solution for cryoprotection, frozen on dry ice and cryosectioned 40 μm in the coronal plane. For identification of astrocytes, a primary anti-glial fibrillary acidic protein antibody (anti-GFAP, #G9269, 1:200, Sigma-Aldrich) and for identification of microglia the Iba-1 antibody (anti-iba-1, ab5076, 1:200, Abcam) was used. The secondary antibodies were Alexa 488 anti-rabbit (1:1000, Molecular Probes, Invitrogen) and Alexa Fluor 594 (1:1000, TermoFisher Scientific), respectively. The slides were sealed with mounting medium containing DAPI (Vector Laboratories, Inc. H-1200) and coverslipped. Three consecutive brain sections, 100 μm apart, were imaged using a Nikon Eclipse 80i microscope and 40× magnification at the level of dorsal hippocampus – anterior thalamus. Four to six rectangular 60 × 60 μm regions of interest were randomly placed on the cerebral cortex bilaterally. The number of reactive astrocytes and activated microglia, identified using morphological criteria^22^ on immunostaining and with nuclei co-localization on DAPI stain (Figure 7), were counted and averaged between sections and region of interest, separately for ipsilateral and contralateral to H-I injury site.

### 4.8. Histological assessment of intestinal injury

After euthanizing pups on P13, 1 cm samples of terminal ileal tissue were harvested, rinsed with saline, and fixed in 4% PFA. Coded frozen cross-sections (20 μm thick) were stained with hematoxylin and eosin by standard protocols. Lamina propria thickness in crypts, villi length and density per ileum cross-section circumference was measured by micrometry under 10×magnification in a blinded manner; at least 10 measurements from non-overlapping well oriented areas were taken from each sample. Five samples were taken from the Fresh water sham group and all microbiota groups after H-I.

### 4.9. Statistical analysis

Effects of microbiome treatment on brain injury volume on MRI, cytokines and TLRs gene expression was tested using a one-way ANOVA, followed by post-hoc Tukey-Kramer group comparisons as implemented in the Matlab R2018a (Natick, MA) statistical toolbox. A two-way ANOVA was used to test the main effects of microbiota group and H-I factors and their interactions on cytokines expression. An ANCOVA was used to test significance of microbiota group on cytokines gene expression (y) with H-I injury volume at 24 h as a covariate (x), using a model y = (α + α_i_) + βx + ε, where αi was the intercept for each microbiota group. Distribution of injury severity outcomes between microbiota treatment groups was calculated using a Chi-squared and Fisher’s exact test in Matlab.

## Supplementary Material

Supplemental Tables clean.docx

## Data Availability

All sequencing data are available on NCBI (bioproject ID: PRJNA1001342).
