# Supplementary material for: Intestinal microbiota modulates neuroinflammatory response and brain injury after neonatal hypoxia-ischemia
Source: Gut Microbes. 2024 Mar 27;16(1):2333808. doi: 10.1080/19490976.2024.2333808 (PMC10978030; doi:10.1080/19490976.2024.2333808)
Supplement: Supplemental Tables clean.docx [file KGMI_A_2333808_SM1146.docx]

*Supplementary Table 1. ANCOVA coefficients estimates for the expression of pro- and anti-inflammatory cytokines dependence on microbial groups with H-I brain injury volume as a covariate. The cytokines gene expression were modeled as y = (α + αi) + βx + ε, where αi was the intercept for each microbiota group with H-I injury volume at 24h as a covariate (x).*

|  | *Intercepts for Microbiota groups (p-value)* | | | | *Slope and p-value for H-I injury volume* |
| --- | --- | --- | --- | --- | --- |
|  | *Fresh water* | *Antibiotics* | *E. coli* | *B. infantis* |  |
| IL1β | ***0.37 (0085)*** | ***-0.58 (0.011)*** | ***0.89 (0001)*** | ***-0.68 (0.009)*** | ***0.194 (0.0016)*** |
| TNFα | ***2.74 (<0.001)*** | ***-2.12 (0.004)*** | *0.72 (0.38)* | ***-1.34 (0.09)*** | ***0.070 (0.0003)*** |
| IL6 | ***0.46 (0.003)*** | *-0.28 (0.14)* | ***0.48 (0.03)*** | ***-0.76 (0.009)*** | *-0.0012 (0.81)* |
| IL2 | ***0.82 (0.0003)*** | ***-0.7 (0.002)5*** | *0.30 (0.24)* | *0.39 (0.12)* | *0.0003 (0.96)* |
| TGFβ | ***0.30 (<0.001)*** | *0.017 (0.76)* | ***-0.31 (<0.001)*** | ***-0.31 (<0.001)*** | ***0.0052 (0.001)*** |
| IL10 | ***0.50 (<0.001)*** | *-0.162 (0.14)* | *0.096 (0.94)* | ***-0.35 (0.005)*** | ***0.0087 (0.0036)*** |

*Supplementary Table 2. Two-way ANOVA results for TLR gene expression in pups’ brains with the main effects of H-I and Microbiota treatment factors and their interactions.*

|  | Main effect:  Microbiota, F(3,107) | p-value | Main effect:  H-I,  F (1,107) | p-value | Interaction:  Microbiota * H-I, F (3,107) | p-value |
| --- | --- | --- | --- | --- | --- | --- |
| TLR2 | 3.20 | **0.026** | 23.52 | **4.2054e-06** | 3.30 | **0.023** |
| TLR3 | 12.04 | **7.4492e-07** | 93.18 | **3.1120e-16** | 3.73 | **0.013** |
| TLR4 | 21.94 | **3.7622e-11** | 26.13 | **1.4032e-06** | 0.95 | 0.41 |
| TLR6 | 4.68 | **0.0041** | 29.45 | **3.5928e-07** | 4.92 | **0.003** |

*Supplementary Table 3. Experimental groups and sample numbers.*

| Microbiota | P10, no injury | P13 carotid ligation +hypoxia | P13,no H-I injury | P13, with injury | Mortality | Total H-I | Mortality, % | P13, global hypoxia | Total |
| --- | --- | --- | --- | --- | --- | --- | --- | --- | --- |
|  |  |  |  |  |  |  |  |  |  |
| Fresh water | 7 | hypoxia | 1 | 16 | 3 | 20 | 15.00% | 4 | 31 |
| Antibiotics | 18 | hypoxia | 4 | 14 | 4 | 22 | 18.18% | 4 | 44 |
| E.coli | 7 | hypoxia | 2 | 13 | 2 | 17 | 11.76% | 4 | 28 |
| B.infantis | 7 | hypoxia | 2 | 8 | 2 | 12 | 16.67% | 4 | 23 |
| Fresh water |  | sham/hypoxia | 0 | 6 | 0 | 6 | 0.00% |  | 6 |

*Supplementary Table 4. Primers used for PCR analysis from Integrated DNA Technologies.*

Cat No. Primer Number/Assay # Name

289446873 Mm.PT.58.45820113 TLr2

289446876 Mm.PT.58.8085919 TLr3

289446879 Mm.PT.58.41643680 TLr4

289446882 Mm.PT.58.42866304.g TLr6

289446885 Mm.PT.58.41769240 IFNG

289446888 Mm.PT.58.41616450 IL1b

289446891 Mm.PT.58.11478202 IL2

289446894 Mm.PT.58.10005566 IL6

289446897 Mm.PT.58.9981538 IL8

289446900 Mm.PT.58.13531087 IL10

289446903 Mm.PT.58.12575861 TNG

289446906 Mm.PT.58.8169936 Ltbp1

291561036 Mm.PT.39a.1 Gapdh
